# Development and preliminary evaluation of a structured and personalized self-help smoking cessation program: A prospective observational study

**DOI:** 10.18332/tpc/208808

**Published:** 2025-09-18

**Authors:** Laure Fillette, Isabelle Varescon

**Affiliations:** 1 Laboratory of Psychopathology and Health Processes, Université Paris Cité, Boulogne, France

**Keywords:** smoking, cessation, behavioral, intervention, self-help, program

## Abstract

**INTRODUCTION:**

We have developed the ‘Two Weeks to Quit’ (TWTQ) program, a self-help smoking cessation toolkit. Self-help programs represent a cost-effective and accessible option for successful smoking cessation. TWTQ includes a two-week preparation phase leading up to a quit-smoking date at the end of week two and a four-week period focused on maintaining a smoke-free status. The objective was to assess its effectiveness.

**METHODS:**

This prospective observational study, conducted without a control group, evaluated the TWTQ program among smokers aged 18–60 years in Paris, France, between February 2023 and April 2024. Participants were recruited via pharmacies, social media platforms, and through outreach to the general population. Program adherence was monitored weekly by email. The primary outcome was smoking cessation, assessed at the end of the program using the Fagerström test for nicotine dependence and a self-reported smoking status question, corresponding to one month after the theoretical quit date.

**RESULTS:**

Of the 97 participants enrolled, 47 completed the program. At six weeks, 57.4% reported smoking cessation, all on the scheduled quit date. At five months, 40.4% of these participants remained abstinent, with an intent-to-treat abstinence rate of 19.6%. The mean Fagerström score decreased significantly from 4 to 0.87 at six weeks (p<0.001), with sustained reductions at two and five months. Adherence to the step order, pacing, and older age significantly predicted short-term cessation. In contrast, no factor predicted abstinence at five months, and greater use of customizable tools was unexpectedly associated with higher relapse risk.

**CONCLUSIONS:**

TWTQ demonstrates potential benefits as a structured self-help program for smoking cessation and tobacco consumption reduction, with sustained effects. Results underscore the need to evaluate both engagement and effectiveness in large-scale campaigns like ‘Stoptober’.

## INTRODUCTION

Over recent decades, self-help smoking cessation programs have emerged as practical and scalable solutions to overcome the logistical, financial, and geographical limitations of traditional clinical interventions^1^. Self-help programs are accessible^2-5^ and cost-effective^6-8^. A 2019 Cochrane review^9^ confirmed their relative effectiveness, particularly when implemented without additional support. The success of these programs is often attributed to participant engagement^10-12^, as smokers benefit from active and engaging elements, such as personalized feedback^1^, to fully leverage these interventions^13,14^. For instance, Brendryen and Kraft^15^, who demonstrated the effectiveness of their ‘Happy Ending’ program, noted that enhanced engagement could further improve results. Conversely, the limited success of digital self-help interventions, like the ‘SmokeFree Buddy’ app, has been attributed to insufficient user engagement^16^. Additionally, it was emphasized that frequent usage is key to the effectiveness of combined mobile applications and cognitive-behavioral therapy, underscoring engagement as a decisive factor^17^.

This raises a fundamental question: ‘What makes a self-help program truly engaging?’. It was found that extensive use of the internet was not associated with higher success rates in smoking cessation^3,5^ and several online programs showed limited results due to their inability to generate sufficient user engagement to ensure sustainable cessation^18-20^. These findings raise questions about the true utility of internet-only interventions as engagement goes far beyond the mere time spent online and is more closely tied to the quality of interaction with program content^11^. Structured programs, which guide participants to actively follow the steps of smoking cessation, promote user engagement by imposing a consistent framework that encourages adherence and allows participants to systematically address their smoking habits^15^. These programs provide a guided framework with limited autonomy, following a precise schedule, enabling users to adapt effectively to the challenges of smoking cessation and engage more actively in the process^2^. Numerous structured programs have proven to be more effective than less structured interventions^1,12,15,21^. The ‘Happy Ending’ (HE) program, which includes a 14-day preparation phase (featuring daily emails and links to a website) followed by a 30-day active phase focused on coping strategies, was significantly more effective compared to an unstructured control intervention, with results sustained up to 12 months post-intervention^2^. Similarly, the ‘Quit For Life’ (QFL) program demonstrated its effectiveness with a two-phase approach: reduction and relapse prevention, spread over 10 sessions across 3 months^21^. Compared to less structured interventions, the QFL program showed higher success rates six months post-intervention, reinforcing the value of structured approaches in supporting long-term smoking cessation. Among these, cognitive-behavioral therapy-based models such as Mindfulness^21^, and Acceptance and Commitment Therapy (ACT)^22^, have also demonstrated their effectiveness^23,24^. ACT stands out for its structured, incremental format, which fosters user engagement and helps address the psychological challenges associated with quitting^11^.

In contrast to structured approaches, personalization aligns programs with the individual needs and preferences of participants. Smokers often find personalized interventions appealing and a Cochrane review identified personalization as an important factor in the effectiveness of self-help smoking cessation programs^9,25^. However, this appeal does not always translate into superior outcomes, especially when personalized self-help programs prioritize user preferences over structured frameworks. For example, the effectiveness of personalized programs may decrease when used alongside nicotine replacement therapies^9^. Moreover, the literature is not unanimous regarding the role of personalization, while some personalized self-help programs demonstrate notable effectiveness compared to non-personalized approaches^1,2,8,12,21,24,26^, others do not offer significantly superior results^4,16,20^. This inconsistency may stem from the diverse interpretations of personalization^27^, which can involve tailoring programs to specific ethnicities^12,19,24^, age^5,10,20^ or individual preferences^4,16^. These variations introduce numerous confounding variables, complicating result comparisons and analyses. For instance, it was found that socioeconomic status influenced abstinence outcomes only in conjunction with ethnic background, suggesting that smoking cessation programs should address both racial and socioeconomic differences^26^. Such findings indicate that while personalization can enhance user engagement, it should not compromise the structural elements that are critical for program effectiveness.

Given these complexities, structured programs emerge as a more consistent and reliable approach. Not only do these programs enhance engagement, but they also facilitate their evaluation and allow for meaningful comparisons with other interventions. Diverse confounding variables usually complicate the evaluation of smoking cessation programs and factors such as access to additional resources (e.g. websites or emails) or the use of nicotine replacement therapies (NRT) often influence outcomes. For example, a study showed that even when participants were encouraged to avoid NRTs, 24% of their intervention group used them, thereby affecting the analysis of program efficacy^2^. Similarly, the use of electronic cigarettes could positively influence cessation rates, adding another layer of complexity to outcome interpretation^11^. Structured programs, by providing a clear and step-by-step framework, allow for more precise evaluation of nicotine substitute use at each stage of the cessation process. By combining enhanced engagement with methodological structure, these programs may help address common challenges such as attrition, confounding variables, and inconsistencies in evaluation, making them a valuable option in the field of smoking cessation^15^. Based on this evidence, we developed the ‘Two Weeks to Quit’ (TWTQ) program, a self-help smoking cessation intervention that integrates structured guidance with elements of personalization to optimize engagement and effectiveness. Developed in line with existing evidence on structured interventions, TWTQ proposes a step-by-step framework intended to support participants in addressing psychological and behavioral challenges related to smoking cessation. Its effectiveness was explored through a prospective observational study assessing its impact on smoking behavior and the factors potentially influencing outcomes. The study was based on two main hypotheses: 1) The TWTQ program would lead to sustained smoking cessation or, at minimum, a long-term reduction in tobacco consumption; and 2) Adherence to the program’s chronological structure and engagement with its tools would be predictive factors of its effectiveness.

## METHODS

### Intervention: description of the program

**Figure 1:**
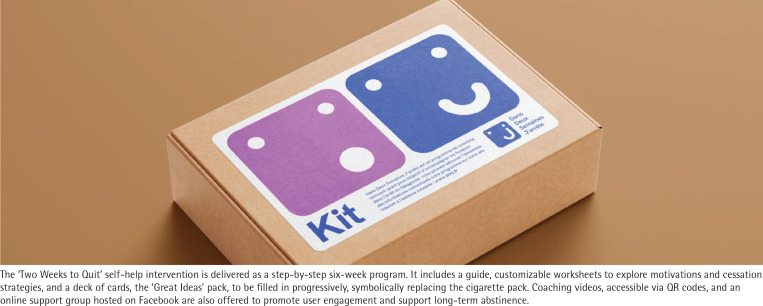
The ‘Two Weeks To Quit’ program kit, prospective observational study, Paris, France, February 2023 to April 2024 (N=97)

The TWTQ program is a six-week hybrid self-help smoking cessation intervention designed to enhance user engagement through a combination of structured planning, cognitive-behavioral strategies, and personalization. Its design was informed by core principles of cognitive psychology, particularly regarding motivation, self-regulation, and habit change. These principles guided the integration of preparatory steps, repeated actions, and self-monitoring components, with the aim of supporting gradual behavioral transformation. Delivered as a comprehensive kit (Figure 1), it includes a printed instruction booklet, step-by-step guides, customizable worksheets, a timeline calendar, advice sheets, and ‘Great Ideas’ cards for recording essential strategies and reflections. These cards are gradually filled in and inserted into a compact box that mimics the dimensions of a cigarette pack, serving as a symbolic and practical replacement for cigarettes throughout the program.

**Figure 2:**
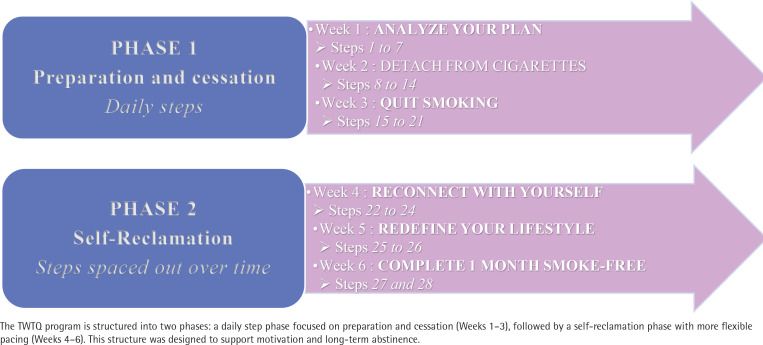
Structure of the ‘Two Weeks to Quit’ program: Two-phase progression over six weeks, prospective observational study, Paris, France, February 2023 to April 2024 (N=97)

Digital resources complement the physical materials, with QR code-accessible coaching videos created by experts in psychology, dietetics and cognitive-behavioral therapy. These videos are designed to provide users with professional guidance and motivational support at each stage of their journey. Participants also have access to a private Facebook support group, moderated by the lead researcher, fostering peer encouragement and shared experience throughout the process. The program follows a structured timeline of 28 steps, always starting on a Monday to align with the natural weekly rhythms known to support the establishment of new healthy habits^28^ and is divided into two distinct phases (Figure 2). By integrating symbolic tools, structured guidance, therapeutic content, and social support, TWTQ offers a multidimensional, user-driven approach to quitting smoking and maintaining long-term behavioral change.

### Study population

Adults aged 18–60 years, French-speaking, and residing in Paris, France, who self-identified as regular smokers wishing to quit tobacco use, and who had access to a smartphone, an internet connection, and a Facebook account, were eligible for inclusion. Pregnant women and individuals with polyaddictions were not included. Participants who withdrew before the end of the study or did not complete the program evaluations were excluded from the analysis. Written informed consent was obtained from all participants prior to inclusion.

Recruitment was carried out primarily through pharmacies and on Instagram, via calls for participation over a 14-month period, from February 2023 to April 2024. Pharmacies were specifically targeted based on the assumption that smokers motivated to quit would go there to purchase nicotine replacement products, as they do during the ‘*Moi(s) sans tabac*’ campaign^29^ – a national public health initiative in France encouraging smokers to quit collectively each November – when they pick up quit kits.

### Measurement tools

At baseline, participants completed a sociodemographic questionnaire including seven items. They were asked to indicate their gender (female, male, or other), their age in years, their current marital status (single, cohabiting, married, in a civil union, divorced or separated, or widowed), and their highest level of education (secondary education, high school diploma or equivalent, Bachelor’s degree, Master’s degree, or other). They were also asked to report their employment status (employed, unemployed, on medical leave, retired, student, or other), their current housing situation (tenant, owner, hosted by someone else, living in a shelter, or homeless), and their native language. All responses were treated as categorical variables, except for age, which was treated as a continuous variable. No grouping or recoding was applied in the analysis.

Participants also completed the Fagerström test for nicotine dependence (FTND)^[Bibr CIT0030]30^, a 6-item instrument used to assess the intensity of physical nicotine dependence. The total score ranges from 0 to 10 and was treated as a continuous variable in the main analyses. For descriptive purposes, the following categories were used: very low dependence (0–2), low (3–4), moderate (5), high (6–7), and very high (8–10).

The Hospital Anxiety and Depression Scale (HADS)^[Bibr CIT0031]31^ was used to assess psychological symptomatology. It consists of 14 items divided into two subscales – anxiety and depression – each ranging from 0 to 21. Following standard interpretation thresholds, scores were categorized as follows: 0–7 indicating no symptomatology, 8–10 suggesting borderline or doubtful symptomatology, and ≥11 reflecting clear symptomatology. These subscale scores were treated as continuous variables in the analyses.

Motivation to quit smoking was assessed with a single Likert-scale item ranging from 1 (‘not at all motivated’) to 5 (‘extremely motivated’), which was treated as a continuous variable. Participants were also asked to report the number of previous quit attempts, recorded as a continuous variable.

A weekly questionnaire was sent to participants during the six-week program. This questionnaire was created specifically for this study and has not undergone prior psychometric validation. It included self-reported smoking status with the exact quit date when applicable, as well as binary questions on whether they had followed the order and pacing of the program steps during the past week. It also included binary items on the use of nicotine replacement therapies and electronic cigarettes, with open-ended responses specifying the type of product, the nicotine dosage, and the frequency of use. Engagement with key program components – customizable tools, exercises, QR-code coaching videos, and participation in the Facebook group – was measured using 5-point Likert scales (from ‘never’ to ‘very often’). Participants were also asked to rate their level of anxiety on a 0–10 scale, and were invited to provide personal remarks about their experience each week.

### Procedure

Participant data were collected at baseline, weekly during the six-week program, at the end of the program, then again two months later (i.e. three months after the theoretical quit date) and five months later (i.e. six months after the theoretical quit date). Weekly assessments were conducted every Sunday during the program. The questionnaire was sent by email each Sunday, with a reminder the day before (Saturday). In the event of non-response, up to two additional email reminders were sent. These weekly data allowed us to analyze participant engagement and adherence to the program timeline (step order and pacing). At the end of the program, all participants completed the Fagerström test for nicotine dependence (FTND) and answered the question ‘Have you quit smoking?’ to report their smoking status. Abstinence was defined as a ‘yes’ response to this question combined with an FTND score of 0. Since some individuals may report a score of 0 on the FTND despite continuing to smoke occasionally, we took care to distinguish abstinent participants from non-abstinent participants with a zero score. For this reason, only FTND scores of 0 reported by participants who answered ‘no’ to the abstinence question were included in longitudinal analyses of tobacco consumption. This approach ensured that changes in nicotine dependence could also be evaluated among participants who did not achieve full abstinence, while clearly excluding abstinent profiles from the analysis of consumption levels.

For the purpose of analysis, participants were classified into two groups based on their level of participation. The ‘Respondents’ group included individuals who completed the full six-week program, responded to the weekly assessments, and completed the post-program FTND. The ‘Non-respondents’ group included all other participants, including those who discontinued the program before completion (dropouts) as well as those who failed to respond to the weekly assessments or post-program evaluation. This classification was used to compare baseline characteristics and program outcomes.

### Statistical analysis

Data were analyzed using SPSS software. Descriptive statistics were used to characterize the sample and adherence patterns. Differences between participants who completed the program and those who did not were assessed using independent samples t-tests for continuous variables. A multiple logistic regression analysis was conducted to identify predictors of smoking cessation, including adherence to the program timeline and engagement with the program’s tools. In addition, paired samples t-tests were used to assess changes in FTND scores over time (baseline, end of program, two months, and five months post-intervention). Correlational analyses (Pearson’s *r*) were conducted to explore associations between engagement with individual tools and changes in FTND scores. Two multiple linear regression analyses were also conducted: the first to identify predictors of short-term reduction in FTND score at the end of the program, and the second to assess whether engagement with program tools predicted the maintenance of FTND score reduction at five months. A significance threshold of p<0.05 was applied to all tests.

## RESULTS

### Participants

The initial sample included 97 participants, the majority of whom were women (53.6%), held a postgraduate degree (62%), and were employed (68%). The mean age was 38 years (SD=12.5, range: 19–61). Overall, motivation to quit smoking was high, with 58% of participants reporting being highly (4/5) or very highly motivated (5/5). Nicotine dependence, measured using the Fagerström score, indicated a moderate average level of dependence (mean score=4, SD=2.4). More specifically, 32% of participants were moderately dependent (score 5–6), 28% were mildly dependent (score 3–4), 28% were non-dependent (score 0–2), and 12% were highly dependent (score ≥7). Participants reported an average of three previous quit attempts, with considerable interindividual variability (range: 0–20). Psychologically, 58% showed signs of anxiety or suspected anxiety disorders, and 17% reported depressive symptoms (Table 1).

**Table 1:** Descriptive statistics collected at baseline and end of the program (Session 0 and Session 1) in a prospective observational study conducted in Paris, France, February 2023 to April 2024 (N=97 at baseline, N=47 at follow-up)

	*Age (years)*	*Motivation*	*Previous attempts*	*HADS result anxiety*	*HADS result depression*	*Fagerström* *result* *nicotine* *dependence*
**Session 0** (N=97)	38.0 (12.5)	3.97 (0.7)	2.90 (3.3)	8.91 (3.67)	4.86 (3.13)	4.00 (2.36)
**Session 1** (N=47)	37.0 (10.6)	4.00 (0.63)	3.32 (4)	8.89 (3.65)	4.32 (2.14)	0.872 (1.65)

Data are given as Mean (SD). Session 0 (S0): data collected for all participants (N=97), including those who did not complete the weekly evaluations and therefore did not complete the Fagerström test at the end of the program. Session 1 (S1): data collected for participants who continued the follow-up (N=47), completed the weekly evaluations, and took the Fagerström test after the program. Motivation to quit smoking was self-rated on a scale from 0 (no motivation) to 5 (very strong motivation). Previous quit attempts are reported as absolute counts. HADS: Hospital Anxiety and Depression Scale.

### Participant typology based on study engagement

A comparison of baseline data revealed several differences between the two groups. Respondents (N=47) showed a slightly lower nicotine dependence at baseline (Fagerström score=3.5) than Non-respondents (N=50; mean score=4.3), but this difference was not statistically significant (p=0.293). They included a higher proportion of women (66.0% vs 53.6%), though this difference was also non-significant [χ^2^(1)=1.43, p=0.232]. However, education level was significantly higher among Respondents (80.9% with postgraduate education vs 62.0%; χ^2^(1)=4.16, p=0.041). Respondents had made more previous quit attempts (mean=3.32 vs 2.90), but this difference was not significant (p=0.425). Anxiety and depression scores were slightly lower in Respondents (HADS anxiety: 8.89 vs 8.91, p=0.975; HADS depression: 4.32 vs 4.86, p=0.359). Motivation to quit was similar in both groups (4.00 vs 3.97, p=0.895).

### Engagement with program tools and adherence to the timeline

Respondents engaged with the program’s tools throughout the six-week period, although their engagement declined over time. Completion of the exercises was reported by nearly 70% of participants in week 1, but gradually decreased to 30% by week 6. Video usage, which was high at the beginning (75% of participants watched them), followed a similar trend, with 30% still watching them by week 4 and 70% having stopped by week 6. The customizable tools were actively used at the start of the program (53% usage in week 1, average score=2.2) and remained in use by a quarter of participants in week 6 (25%, average score=0.9). The Facebook group was rarely utilized from the outset (87% had never accessed it in week 1), and its use remained marginal at the end of the program (95% had never accessed it in week 6). As for adherence to the program steps, 66% of participants were still following the order of the instructions in week 6, compared to 91% in week 1, reflecting a relative maintenance of program structure. The pacing of the steps showed consistent stability throughout the six weeks (56% in week 1; 52% in week 6), suggesting that participants followed the program at their own rhythm without significant fluctuation

### Effects of the program on respondents’ smoking status

The ‘abstinent’ smoking status at the end of the program was defined by a score of zero on the FTND and a ‘yes’ response to the question ‘Have you quit smoking?’. Among the 47 Respondents, 27 (57.4%) reported being abstinent at the end of the program. All abstinent participants indicated a quit date that matched the one planned by the program, namely two weeks after the start of the intervention. At follow-up, 23 participants (48.9%) remained smoke-free two months after completing the program, and 19 (40.4%) maintained abstinence five months after the program ended. When analyzed using an intent-to-treat approach – considering all non-respondents as smokers – the five-month abstinence rate was 19.6% (19 out of 97 participants). Although this is a more conservative estimate, it reflects a realistic measure of the program’s effectiveness in routine conditions and supports its relevance as a scalable self-help intervention.

### Predictive factors of smoking cessation and maintenance

A logistic regression analysis was conducted to identify the predictors of immediate smoking cessation at the end of the program. Three factors significantly predicted abstinence: adherence to the prescribed step order (p=0.032), adherence to the recommended pace (p=0.043) and participants’ age (p=0.047), with older participants being more likely to succeed. In contrast, the use of customizable tools, exercises, videos, the Facebook group, or anxiety levels were not significantly associated with short-term cessation. To explore whether the effects of the program were maintained over time, a multiple regression was used to assess the predictive value of engagement with the program’s tools on abstinence at five months. None of the predictors – use of customizable tools, exercises, videos, or the Facebook group – were significantly associated with smoking status at five months. The regression model was not significant (R^2^=0.183; p=0.246), suggesting that long-term outcomes may be influenced by factors outside the program’s scope, such as intrinsic motivation or social environment. Interestingly, a counterintuitive finding emerged: higher engagement with customizable tools in the early weeks was associated with a greater likelihood of relapse at five months (p=0.024). This may reflect a compensatory effort among participants experiencing more difficulty with quitting.

### Changes in tobacco consumption among non-abstinent respondents

In parallel with the assessment of abstinence, we examined whether the program also had a measurable effect on tobacco consumption among participants who did not quit smoking entirely. The objective was to determine whether a significant reduction in tobacco consumption could be observed, even in the absence of full abstinence. To assess this, we used the FTND, administered at four time points: before the program (baseline), immediately after completion, and at two and at five months post-program. At baseline (Session 0), Respondents (N=47) had a mean FTND score of 3.5 (SD=2.36), which was slightly lower than the total sample (mean=4.28, SD=2.37). After six weeks in the program (Session 1), the mean score among Respondents had decreased to 0.87 (SD=1.65), representing a statistically significant reduction [t(46)=7.71, p<0.001], with a large effect size (Cohen’s d=1.12). This suggests a strong impact of the program on reducing tobacco consumption, regardless of abstinence status. Beyond these immediate effects, we conducted a longitudinal analysis to determine whether the reduction in tobacco consumption was maintained over time (Table 2). The mean FTND score remained very low at the follow-up at two moths (mean=0.39, SD=0.87), and although a slight increase was observed at five months (mean=1.03, SD=2.04), it remained substantially below baseline levels. Paired-sample t-tests confirmed that the reduction was statistically significant both at two months [t(35)=7.94, p<0.001] and at five months [t(30)=4.52, p<0.001], with no significant difference between the post-program score and the follow-up scores. These findings suggest that the program’s effects on tobacco consumption were not only immediate but also sustained. Participants appeared to have internalized the benefits of the intervention in a durable way, with no significant rebound in consumption scores during the five-month study period.

**Table 2:** FTND scores at four time points, prospective observational study of smokers aged 18–60 years, Paris, France, February 2023 to April 2024

*Measurement time point*	*N*	*Mean*	*SD*	*Median*	*Range*
Before the program	47	3.49	2.18	3	0–9
Immediately after the program	47	0.87	1.65	0	0–6
Two months after	36	0.39	0.87	0	0–3
Five months after	31	1.03	2.04	0	0–6

FTND: Fagerström test for nicotine dependence.

### Predictive factors of the FTND score reduction

A multiple linear regression analysis was conducted to identify the predictors of reduction in tobacco consumption, measured by the difference in Fagerström scores between baseline (Session 0) and the end of the program (Session 1). The dependent variable was a continuous score (ΔFagerström=S1-S0), where higher negative values indicated a greater reduction in consumption. The overall model explained 24.1% of the variance in consumption reduction (R^2^=0.241). Adherence to the prescribed order of program steps was significantly associated with a greater a greater consumption reduction (β=0.593, p=0.003; 95% CI: 0.211–0.976). In contrast, adherence to the pacing of the steps was not a significant predictor (β=0–0.160, p=0.402; 95% CI: -0.542–0.222). The use of customizable tools was also a significant predictor of consumption reduction (β=0.681, p=0.002), whereas the use of exercises (β= -0.072, p=0.766) and video content (β= -0.148, p=0.326) had no significant effect. Finally, the use of the Facebook group remained marginal and was not associated with any measurable impact on consumption scores.

### Predictive factors for long-term effectiveness

To explore which factors might predict the maintenance of consumption reduction at five months post-program, a multiple linear regression was conducted. The aim was to assess whether engagement with program tools could predict Fagerström scores at the follow-up at five months. The model included four predictors: Facebook group usage, customizable tools usage, exercise completion, and video viewing. The dependent variable was the Fagerström score at five months, and the analysis was conducted on the 31 participants with available follow-up data (Table 3). The overall model was not statistically significant [F(4, 26)=1.45, p=0.246, R²=0.183]. Customizable tool usage, which had previously predicted short-term reduction, was not associated with Fagerström scores at five months. Similarly, no significant associations were found for the use of exercises, videos, or the Facebook group.

**Table 3:** Multiple linear regression predicting FTND scores at five months, prospective observational study, Paris, France, February 2023 to April 2024 (N=31)

*Variable*	*β*	*t*	*p*	*95% CI*
Intercept	2.94	3.05	0.005	0.96–4.92
Facebook group usage	0.03	0.17	0.868	-0.37–0.44
Customizable tools	0.07	0.67	0.511	-0.14–0.28
Exercise completion	-0.17	-1.47	0.155	-0.40–0.07
Video viewing	-0.04	-0.46	0.649	-0.21–0.13

Linear regression model assessing whether use of program tools (Facebook group, customizable tools, exercises, and videos) predicted FTND scores five months after completing the TWTQ program.

Additionally, a logistic regression was conducted to assess predictors of abstinence status at five months (abstinent vs non-abstinent). The model included engagement variables, anxiety level, and age. None of the predictors reached significance, though one effect was notable: higher use of customizable tools was significantly associated with a greater likelihood of relapse (p=0.024). Age also approached significance (p=0.071), suggesting a possible trend toward higher abstinence among older participants.

### Use of nicotine replacement therapies and electronic cigarettes

No participant was using nicotine replacement therapy (NRT) or electronic cigarettes (EC) at the time of inclusion, and none had initiated a quit attempt prior to starting the program. General guidance on the use of cessation aids was provided as part of the program, including encouragement to seek advice from a pharmacist. Participants were also invited to try NRT products during the preparatory phase (the first two weeks of the program) to become familiar with them prior to their potential quit date.

Use of NRT and EC was controlled for in the analysis. Two simple linear regressions were conducted to assess their association with abstinence and reduction in Fagerström scores. Neither NRT use (β=0.29, p=0.379) nor EC use (β=0.13, p=0.494) was significantly associated with smoking reduction or abstinence at the end of the program. These results suggest that the program’s effectiveness was primarily driven by structural and motivational factors rather than pharmacological ones. Consequently, these variables were not included in the multiple regression model. Moreover, the use of NRT and EC was not significantly associated with either sustained abstinence or changes in tobacco consumption assessments at 2 and 5 months.

## DISCUSSION

The TWTQ program demonstrated encouraging results in promoting smoking cessation and reducing tobacco consumption, particularly among participants who actively engaged with the intervention. Among Respondents, the program led to a notable abstinence rate at the end of the six-week period, as well as a significant decrease in tobacco consumption, as measured by the Fagerström test. These outcomes support the potential of TWTQ as a scalable and effective self-help tool.

Logistic regression analyses underscored the importance of structure. Adherence to the prescribed step order significantly predicted reductions in Fagerström scores – interpreted here as a reduction in tobacco consumption – while adherence to the recommended pace had no significant effect on this outcome. This suggests that participants may benefit from some flexibility in pacing, provided the structural sequence of the program is respected.

Customizable tools also emerged as a key component of engagement, predicting short-term reductions in tobacco consumption more robustly than exercises or video content. However, these tools were not associated with smoking cessation per se. In contrast, participants who successfully quit smoking by the end of the program had generally adhered strictly to both the step order and the recommended pace. This dual adherence reflects a more linear and externally guided use of the program, possibly indicating less personal appropriation of the material. Conversely, participants who used the program more flexibly – adapting the tools to their needs and progressing at their own rhythm – tended to reduce their consumption significantly but did not achieve full abstinence. These distinct patterns suggest that strict structure may facilitate cessation, while flexible engagement may support gradual behavioral adjustment in those not yet ready to quit entirely.

This contrast could help clarify the role of personalization, which has been associated with program success in some studies^1,2,8,12,21,24,26^, but not in others^4,16,20^. It is plausible that personalization may assist in initiating change or modifying consumption patterns, without necessarily leading to complete smoking cessation.

The longitudinal follow-up results reinforced the apparent durability of the program’s impact. Two and five months after the end of the intervention, FTND scores remained significantly lower than at baseline, with no evidence of statistical rebound. These sustained reductions suggest that many participants were able to internalize the gains achieved during the program and maintain behavioral changes over time.

However, when abstinence was examined specifically, no engagement-related variable – neither tool use nor structural adherence – was found to predict smoking status at five months. A multiple regression analysis confirmed the absence of significant predictors of long-term FTND score, while a separate logistic regression revealed that greater use of customizable tools during the first weeks was significantly associated with an increased risk of relapse. This counterintuitive result again raises questions about the role of personalization: while flexible engagement with personalized components may help support partial behavioral change, it may also reflect compensatory mechanisms among participants experiencing greater difficulty in maintaining abstinence.

Digital peer support, in the form of the Facebook group, remained marginal throughout the intervention and showed no association with any outcome. This finding aligns with previous research^15–17^ suggesting that digital communities may have limited impact. Future developments could benefit from strengthening peer interaction through structured facilitation and real-time support.

Lastly, to better understand the underlying psychological processes that influence long-term engagement and maintenance, qualitative methods should be included in future research. Exploring subjective experiences and motivations may help refine the intervention, and clarify which elements contribute to lasting behavior change versus short-term improvement.

### Limitations

This study has several limitations that should be acknowledged. First, although the six-week program showed positive outcomes, the follow-up period was limited to five months post-intervention (six months post theoretical cessation date). While this timeframe allows for a medium-term assessment, prior research has demonstrated that cessation outcomes may continue to fluctuate up to one year after the quit date^1^. Therefore, to better evaluate the sustainability of results, future studies should include extended follow-up periods up to 12 months post-intervention.

Second, smoking status and tool usage were self-reported, which introduces the risk of response and recall bias. Biochemical verification (e.g. CO monitoring) and digital tracking of tool engagement would strengthen the reliability of future evaluations by reducing the risk of misclassification associated with self-reported smoking status and tool usage.

Third, the study did not include a control group. As such, it is not possible to establish a causal relationship between participation in the program and the outcomes observed. We can only formulate hypotheses regarding the program’s effectiveness. Furthermore, the results cannot be directly compared to other interventions or to natural quit rates. While the positive outcomes suggest that the TWTQ program may be effective, controlled trials are needed to confirm these findings and isolate the specific contribution of each component of the intervention.

Fourth, the regression model assessing long-term predictors of tobacco consumption did not identify significant relationships with tool usage, possibly due to limited statistical power. This highlights the need for larger sample sizes and more granular measurement tools to understand what drives long-term abstinence.

Additional limitations should be acknowledged. First, the absence of certain covariates in the analyses may have led to residual confounding. Second, participants were primarily recruited through community pharmacies, which may have introduced selection bias. This recruitment method may have attracted individuals who were already more motivated to quit smoking or who had specific expectations about the program, limiting the generalizability of the findings. Third, a progressive decline in participant engagement was observed during the program, raising questions about the factors influencing long-term adherence. Moreover, the use of the FTND to assess changes in tobacco use may itself represent a methodological limitation. Although widely used to evaluate nicotine dependence, the FTND was not originally designed to measure changes in smoking behavior over time, especially among participants who reduce consumption without achieving full abstinence. Future studies could benefit from incorporating complementary measures to better capture nuanced patterns of tobacco use. Finally, the relatively small sample size and geographical specificity of the study (urban area in France) limit the external validity of the results. In addition, if loss to follow-up was not random but associated with both the intervention and smoking outcomes, this could have introduced attrition bias, further challenging the interpretation of long-term effects.

## CONCLUSIONS

The TWTQ program offers a promising model for a structured and user-centered approach to smoking cessation. With a short-term abstinence rate exceeding 57% among respondents and a sustained reduction in tobacco consumption up to five months post-program, it appears to be a viable hybrid solution that blends structure, flexibility, and self-guided engagement. Key strengths of the program include its phased organization, its emphasis on behavioral sequencing, and the integration of therapeutic tools that participants can adapt to their own needs. While engagement with program features declined over time – as is often observed in behavioral interventions – results indicate that sustained participation can lead to meaningful outcomes.

Notably, findings suggest that the type of engagement matters: participants who strictly followed the program structure were more likely to achieve smoking cessation, whereas those who personalized their progression and used the tools more flexibly tended to reduce their tobacco use but were less likely to quit entirely. This distinction may help clarify how personalization operates within self-help interventions: it may be particularly effective in fostering initial change or consumption reduction, but structured adherence appears more strongly associated with abstinence. Future versions of the program could explore ways to balance these two pathways depending on the user’s goal and stage of change.

The absence of predictive power for tool usage at five months highlights the importance of identifying additional mechanisms of support to maintain long-term benefits. These may include extended access to guidance, relapse-prevention strategies, or more actively integrated social support features.

The TWTQ program contributes to the growing body of work on structured, accessible self-help interventions for smoking cessation, particularly those grounded in behavioral theory and psychological insight. While these findings are encouraging, the absence of a control group and the sampling method used prevent any conclusions regarding causality. Further studies, including randomized controlled trials, are needed to confirm the program’s effectiveness and to determine its potential role within broader tobacco control strategies.

## Data Availability

The data supporting this research are available from the authors on reasonable request.
